# Knowledge-guided learning with curated prior genetic biomarkers for robust model interpretation

**DOI:** 10.1093/bioinformatics/btag411

**Published:** 2026-08-21

**Authors:** Beomsu Baek, Eunyoung Jang, Sai Phani Parsa, Youngsoon Kim, Mingon Kang

**Affiliations:** Department of Computer Science, University of Nevada, Las Vegas, NV 89154, United States; Department of Computer Science, University of Nevada, Las Vegas, NV 89154, United States; Department of Computer Science, University of Nevada, Las Vegas, NV 89154, United States; Department of Information and Statistics, Gyeongsang National University, Jinju, Republic of Korea; Department of Computer Science, University of Nevada, Las Vegas, NV 89154, United States

## Abstract

**Motivation:**

Knowledge-guided learning offers effective and robust model training strategies in data-scarce settings by incorporating established domain knowledge, thereby enhancing generalization, robustness, and interpretability. By contrast, conventional deep learning approaches rely purely on data-driven learning, which can limit robust model interpretability, particularly in high-dimensional settings with limited size samples. In computational biology, knowledge-guided learning has primarily leveraged network- and structural-based knowledge, leading to biologically interpretable representations and enhanced predictive performance compared to conventional approaches. However, curated biomarkers, one of the most accessible forms of biological knowledge, remain largely unexplored within knowledge-guided paradigms.

**Results:**

In this study, we propose a model-agnostic training paradigm, Biomarker-driven Explainable Prior-guided Learning (BioExPL), that can be applied to any neural networks that incorporates curated prior knowledge. BioExPL enforces neural networks to reflect curated biomarker priors in their latent representations through a novel knowledge-alignment loss. BioExPL consistently demonstrated significantly improved predictive performance and enhanced model interpretability with minimized computational overhead in simulation studies and intensive experiments on multiple cancer datasets. BioExPL not only integrates prior curated knowledge into the model but also accurately identifies unknown associated signals additionally. BioExPL is model-agnostic and domain-independent, enabling its integration into diverse neural network architectures.

**Availability and implementation:**

The open-source is publicly available at: https://github.com/datax-lab/BioExPL.

## 1 Introduction

Recent advances in artificial intelligence has produced major breakthroughs in computational biology, delivering highly accurate modeling approaches for a broad range of applications, such as disease risk prediction (Zhang *et al.* 2026), survival analysis (Cui *et al.* 2025), and biomarker discovery (Arango-Argoty *et al.* 2025). Deep learning models, in particular, have shown remarkable effectiveness by leveraging highly flexible, data-driven approaches to learn intricate biological mechanisms. Despite the advances in AI, its use in biology is still severely constrained by the scarcity of data. Genomic datasets frequently contain tens of thousands of features measured from only a few hundred patients, which not only degrades predictive performance but also raises concerns about the robustness of models for both prediction and interpretation. Moreover, deep learning models that are trained from scratch face challenges in obtaining reproducible interpretations and frequently fail to identify known biomarkers when interpreting the model.

The knowledge-guided paradigm has emerged as a promising approach to mitigate these challenges. The paradigm incorporates domain knowledge into deep learning models to improve generalization, robustness, and interpretability (Zhou *et al.* 2025). For instance, physics-informed neural networks integrate physical laws into neural network training, resulting in substantial gains in robustness and reliability (Raissi *et al.* 2019). Moreover, a variety of knowledge-guided approaches have been proposed, including constraint-based learning (Lin *et al.* 2025), equation-regularized neural networks (Kuzhiyil *et al.* 2024), and structure-aware representation learning (Xu *et al.* 2025). These studies have demonstrated that incorporation of prior knowledge into deep learning models can markedly enhance both predictive performance and scientific credibility in various scientific domains.

In computational biology, prior knowledge can be broadly categorized into three types: network- and structural-based knowledge and curated biomarkers priors. First, network-based knowledge captures interactions among molecular entities, such as gene–gene and protein–protein interactions, and is commonly incorporated through graph-based modeling. The network-informed learning introduces relational dependencies to the models, facilitating the identification of interaction-driven biological effects (Zitnik *et al.* 2018). Second, structural-based knowledge, such as biological pathway databases and functional modules, were incorporated by constraining neural network architectures. For instance, pathway-informed models embed curated pathway structures directly into model connectivity to enable pathway-level interpretation, while improving robustness and biological plausibility in genomics applications (Hao *et al.* 2018, Elmarakeby *et al.* 2021). Lastly, curated biomarkers priors reduced the high-dimensional spaces of the input data by considering only validated evidence derived from experimental and population-level studies. However, curated biomarker approaches remain largely unexplored within knowledge-guided paradigms.

The most straightforward schemes that leverage curated biomarker priors are feature filtering and feature weighting (Pudjihartono *et al.* 2022). Feature filtering restricts model inputs to a predefined set of curated biomarkers, whereas feature weighting prioritizes curated biomarkers by assigning them greater weights relative to other features. These strategies have been widely adopted in computational biology to constrain high-dimensional molecular profiles to a biologically relevant subspace of characteristics (van Hilten *et al.* 2021, Kim and Lee 2023, Liu *et al.* 2024). However, these schemes have inherent limitations. Penalizing non-curated biomarkers hinders the model in identifying potentially relevant signals that can be associated with the target outcome (Libbrecht and Noble 2015). Relevant biomarkers often appear weak or irrelevant in isolation in complex biological systems, yet they become informative and predictive only when considered in combination with others or within particular biological contexts.

**Figure 1:**
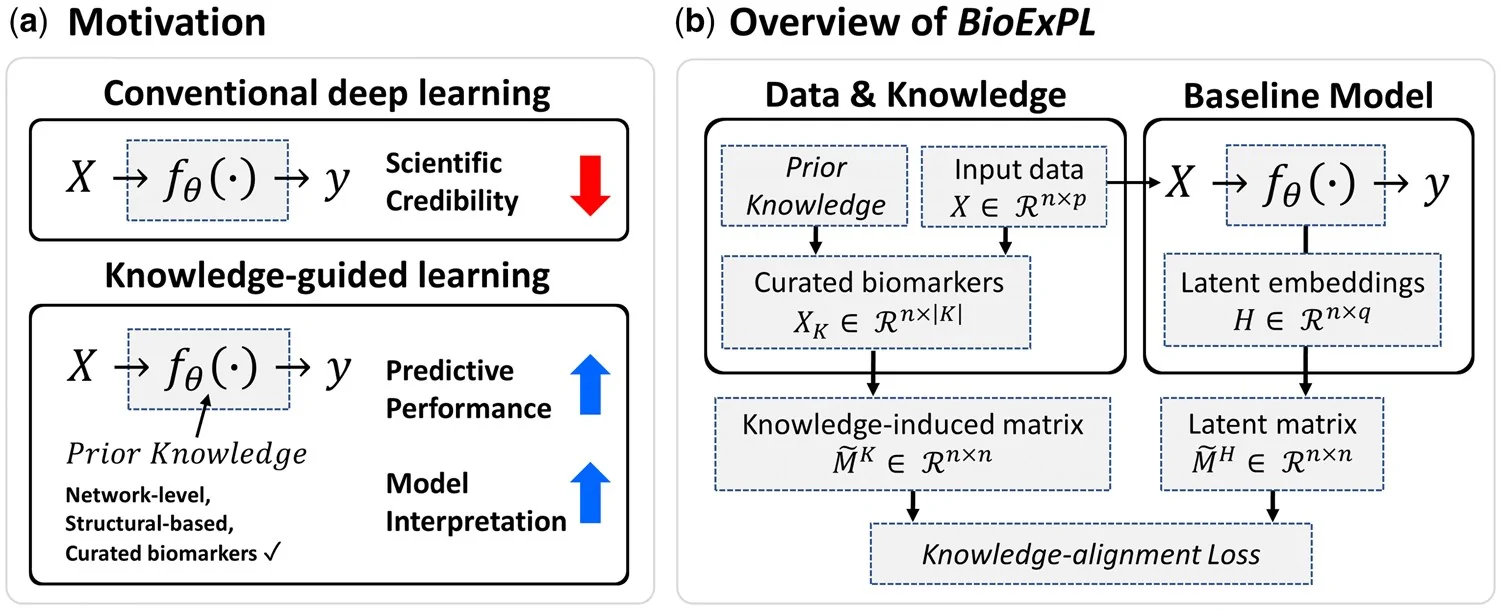
Overview of the study. (a) Motivation. Comparison between conventional deep learning and knowledge-guided learning. (b) Overview of BioExPL. A graphical representation of BioExPL, illustrating how curated biomarkers guide representation learning through a knowledge-alignment loss.

In this study, we propose a model-agnostic training paradigm, Biomarker-driven Explainable Prior-guided Learning (BioExPL), that incorporates curated prior knowledge into neural networks (Fig. 1). BioExPL guides representation learning to reflect specified biologically validated biomarkers to the optimal model by aligning latent embeddings with knowledge-induced representations. This design drives models to embed curated biomarkers into their latent representations, while still allowing the exploration of uncharacterized potential biomarkers, resulting in enhanced predictive performance and robust model interpretation. We validated BioExPL’s performance in a controlled simulation setting and survival analysis experiments with multiple cancer datasets.

## 2 Methods

### 2.1 Objective function for knowledge-guided learning

We present a new knowledge-guided learning approach, named BioExPL, that serves as an effective training strategy by introducing a novel knowledge-alignment loss regularization. Let $X\in R^{n\times p}$ denote the input data matrix, where *n* is the number of samples and *p* is the total number of features. Based on prior biological knowledge, the feature space is conceptually partitioned into two disjoint subsets: the set of knowledge-guided features $X_{K}$, indexed by K⊂{1,…,p}, which are known to be associated with the target outcome, and the set of non-knowledge features indexed by the complement of K, denoted as $X_{\neg K}$.

Let $f_{\theta }(\cdot )$ denote a neural network baseline model with parameters θ, which is trained by minimizing a task-specific loss $L_{\text{task}}$ (e.g. mean squared error or cross-entropy). The objective function of BioExPL combines a task-specific loss with the knowledge-alignment loss ($L_{\text{KAL}}$):


(1) $$L_{\text{total}}=L_{\text{task}}+\lambda L_{\text{KAL}},$$

Where λ≥0 is a hyperparameter controlling the strength of the knowledge alignment. A larger value of λ enforces stronger alignment, thereby driving the model to emphasize knowledge-guided features $X_{K}$. Conversely, a smaller value of λ allows the model to identify potential signals in non-knowledge features $X_{\neg K}$.

### 2.2 Knowledge-alignment loss

The knowledge-alignment loss is formulated by aligning two sample-wise distance or similarity matrices: (1) a knowledge-induced matrix (${\tilde{M}}^{K}$) derived from $X_{K}$, and (2) a latent matrix (${\tilde{M}}^{H}$) computed from intermediate latent embeddings of the neural network. The knowledge-induced matrix serves as a fixed reference that encodes prior knowledge. The latent matrix is optimized to align with the knowledge-induced matrix during training. $L_{\text{KAL}}$ minimizes the sum of squared errors (SSE) between the two matrices:


(2) $$L_{\text{KAL}}=\sum_{(i,j)\in \Omega }{\left({\tilde{M}}_{ij}^{K}-{\tilde{M}}_{ij}^{H}\right)}^{2},$$

Where Ω={(i,j)∣i<j}. ${\tilde{M}}^{K}$ is computed solely from the knowledge-guided features $X_{K}$. Minimizing $L_{\text{KAL}}$ therefore drives the latent embeddings to primarily embed the knowledge-guided features $X_{K}$.

The sample-wise matrices, ${\tilde{M}}^{K}$ and ${\tilde{M}}^{H}$, can be constructed using a range of distance or similarity metrics (e.g. Euclidean distance or cosine similarity). In this study, we adopt Euclidean distance as a representative metric. Given the knowledge-guided feature matrix, $X_{K}\in R^{n\times |K|}$, the knowledge-induced matrix $M^{K}\in R^{n\times n}$ is calculated by the sample-wise Euclidean distance:


(3) $$M_{ij}^{K}={\left\|X_{K}^{\left(i\right)}-X_{K}^{\left(j\right)}\right\|}_{2},\;\forall i,j\in \{1,\ldots ,n\},$$

Where the superscript (i) denotes the *i*-th sample. The latent matrix is derived from an intermediate mapping of the neural network. The intermediate layer can correspond to any hidden layer or an embedding layer of a neural network, $f_{\theta }(X)=g_{\phi }\left(h_{\psi }(X)\right)$ depending on the specific model architecture, where $h_{\psi }(\cdot )$ represents an encoding function up to an intermediate layer and $g_{\phi }(\cdot )$ denotes the remaining task-specific mapping. The latent embeddings are given by


$$H=h_{\psi }(X)\in R^{n\times q},$$

Where *q* denotes the latent dimensionality. Based on the embeddings, the latent matrix $M^{H}\in R^{n\times n}$ is computed as


(4) $$M_{ij}^{H}={\left\|H^{\left(i\right)}-H^{\left(j\right)}\right\|}_{2},\;\forall i,j\in \{1,\ldots ,n\}.$$

To ensure the scale comparability, both matrices are normalized by their respective maximum values:


(5) $${\tilde{M}}_{ij}^{K}=\frac{M_{ij}^{K}}{\max(M^{K})+\epsilon },\;{\tilde{M}}_{ij}^{H}=\frac{M_{ij}^{H}}{\max(M^{H})+\epsilon },$$

Where ϵ is a small constant preventing division by zero. Both $M^{K}$ and $M^{H}$ are distance matrices with a minimum value of zero, resulting in scale-invariant matrices (${\tilde{M}}^{K}$ and ${\tilde{M}}^{H}$) within the range[0,1].

## 3 Experimental results

We evaluated BioExPL through simulation studies and survival analysis using multiple cancer datasets. First, the simulation study assessed whether BioExPL can effectively guide neural networks using predefined knowledge-guided features under controlled settings, where the ground truth of the true associated feature set is known. Second, we applied the proposed BioExPL to real cancer datasets to assess predictive and interpretation performance, comparing with the baseline and benchmark models.

### 3.1 Simulation study

In the simulation study, we designed a synthetic dataset with pre-defined ground truth, where only a subset of features were defined as truly associated features (i.e. $X_{T}$). The simulation study informs only a subset of those truly associated features as knowledge-guided features (i.e. $X_{K}$), instead of guiding all truly associated features, so that BioExPL can discover the un-informed set. Under this setting, we tested the following hypotheses: (1) the proposed knowledge-guided learning strategy, BioExPL, improves predictive performance compared to a baseline model; (2) BioExPL accurately identifies knowledge-guided features ($X_{K}$) more than the baseline; (3) BioExPL effectively suppresses irrelevant features ($X_{\neg T}$); and (4) BioExPL can discover truly associated features that are not included in the predefined knowledge-guided feature set ($X_{T\setminus K}$).

We generated an input data matrix $X\in R^{1,000\times 100}$, where each feature was independently generated from a normal distribution: X∼N(0,1). Among the 100 features, we designated the first five features (i.e. $x_{1}$–$x_{5}$) as truly associated features, and $x_{6}$–$x_{100}$ as irrelevant features. The response variable was generated as a nonlinear combination of the associated features:


(6) $$y=x_{1}^{2}+\text{log}\;\left(|x_{2}|+1\right)+x_{3}x_{4}+\text{sin}(x_{5})+\varepsilon ,$$

Where ε is a noise term that follows a zero-mean Gaussian distribution and was scaled to satisfy a signal-to-noise ratio (SNR) of 10. To ensure comparable effect sizes across the associated features, each nonlinear component in (6) was scaled to have unit variance of mean zero before generating the response variable. Among the five associated features, we designated only the first three features as the knowledge-guided feature set (i.e. $X_{K}=\{X_{1},X_{2},X_{3}\}$). Note that $x_{4}$ and $x_{5}$ were associated features but were not included in the knowledge-guided feature set. $x_{4}$ was designed to interact with the knowledge-guided feature $x_{3}$, and $x_{5}$ was with an independent effect with nonlinear association. We employed a simple multilayer perceptron model (MLP) with three hidden layers as the baseline model and applied BioExPL with the hyperparameter λ varying over $\left\{10^{-3},10^{-2},10^{-1},10^{0},10^{1}\right\}$. To implement the knowledge alignment loss, we computed the latent embeddings (H) from the first hidden layer of the MLP and used Euclidean distance as the pairwise distance metric. We conducted 5-fold cross-validation and repeated the experiment 30 times, reporting the average mean squared error (MSE) across all folds and repetitions.

**Figure 2:**
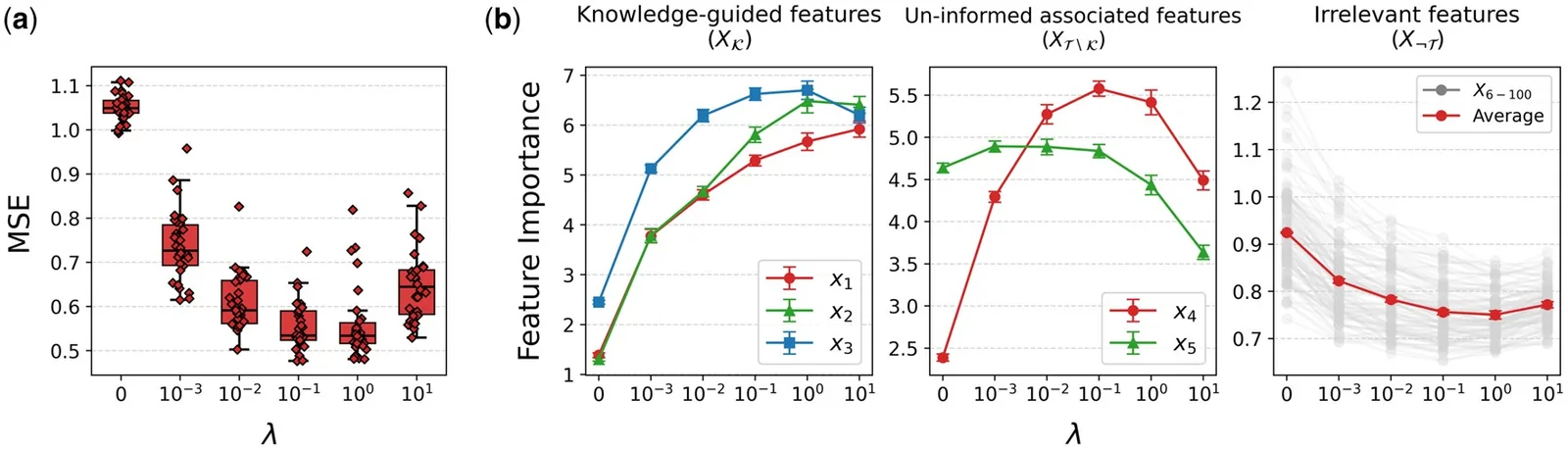
Experimental results on synthetic data. (a) Predictive performance across a range of λ values. Each point represents the average mean squared error (MSE) obtained from 5-fold cross-validation. (b) Feature importance across different values of λ. Feature importance scores were computed using the Integrated Gradients attribution method, and the 1,000 feature importance values were normalized to sum to one to ensure comparability across experiments.

BioExPL consistently improved the predictive performance of the baseline MLP (i.e. λ=0) over varying λ (Fig. 2a). Specifically, BioExPL achieved an average MSE of 0.7374±0.0142, 0.6096±0.0115, 0.5558±0.0099, 0.5592±0.01480 and 0.6491±0.0143 at $\lambda \in \{10^{-3},10^{-2},10^{-1},10^{0},10^{1}\}$, respectively. These results correspond to improvements of 29.7%, 41.8%, 47.0%, 46.6%, and 38.1% compared the baseline MLP (1.0490±0.0054). The improvements were further statistically validated using the Wilcoxon rank-sum test, with P<0.01 for all values of λ.

Furthermore, we analyzed feature importance to investigate whether BioExPL can effectively identify the predefined knowledge-guided features compared to the baseline model. We computed importance scores for each variable using the Integrated Gradients attribution method (Sundararajan *et al.* 2017) for model interpretation. BioExPL effectively emphasized knowledge-guided features while suppressing irrelevant features. With $\lambda =10^{-1}$, which achieved the best predictive performance, the average importance score of knowledge-guided features increased from 1.7135±0.0300 to 5.9084±0.1188 (Fig. 2b, $X_{K}$). In contrast, the average importance score of irrelevant features decreased from 0.9245±0.0015 to 0.7564±0.0053 (Fig. 2b, $X_{\neg T}$).

In contrast to the irrelevant features, we observed that BioExPL successfully identified $x_{4}$ and $x_{5}$ as significant features, although they were not included in the predefined knowledge-guided set (Fig. 2b, $X_{T\setminus K}$). Specifically, BioExPL was more effective at identifying features that exhibit interaction effects with knowledge-guided features. The importance score of $x_{4}$, which interacts with the knowledge-guided feature $x_{3}$, increased substantially from 2.3885±0.0425 to 5.5768±0.0895, whereas the importance score of $x_{5}$ increased modestly from 4.6387±0.0549 to 4.8384±0.0797.

### 3.2 TCGA survival analysis

We conducted survival analysis using cancer gene expression (RNA-seq) data to evaluate BioExPL in a real-world application. We applied BioExPL to several state-of-the-art survival analysis models across multiple cancer datasets from The Cancer Genome Atlas (TCGA). Specifically, we considered ten TCGA cancer datasets, including Liver Hepatocellular Carcinoma (LIHC), Breast Invasive Carcinoma (BRCA), Colon Adenocarcinoma (COAD), Lung Squamous Cell Carcinoma (LUSC), Skin Cutaneous Melanoma (SKCM), Lung Adenocarci-noma (LUAD), Acute Myeloid Leukemia (LAML), Kidney Renal Clear Cell Carcinoma (KIRC), Kidney Renal Papillary Cell Carcinoma (KIRP), and Glioblastoma Multiforme and Lower Grade Glioma (GBM/LGG). In this experiment, we obtained curated cancer-associated genes as the knowledge-guided feature set for each cancer type, from the comparative toxicogenomics database (Davis *et al.* 2025) that provides curated gene–disease associations (https://ctdbase.org/downloads/#c_gd) supported by extensive prior biological literature. The number of curated biomarkers for each cancer type is reported in Table 1, and a brief description of each cancer dataset is provided in Supplementary Note S1, available as supplementary data at *Bioinformatics* online.

**Table 1:** Predictive performance comparison across ten TCGA cancer datasets.

	Cox-nnet				DeepSurv				AESurv			
Dataset (Biomarker #)	Baseline	Filtering	Weighting	BioExPL	Baseline	Filtering	Weighting	BioExPL	Baseline	Filtering	Weighting	BioExPL
**LIHC** (478)	0.6319 (0.0007)	0.6431 (0.0018)	0.6372 (0.0005)	0.6533** (0.0014)	0.6253 (0.0015)	0.6480 (0.0026)	0.6388 (0.0015)	0.6553** (0.0016)	0.6362 (0.0006)	0.6537** (0.0012)	0.6389 (0.0008)	0.6474 (0.0010)
**BRCA** (466)	0.6458 (0.0013)	0.6188 (0.0028)	0.6479 (0.0012)	**0.6499 (0.0011)**	0.6142 (0.0020)	0.6115 (0.0031)	0.6234 (0.0018)	0.6390** (0.0015)	0.6529 (0.0010)	0.6328 (0.0012)	0.6464 (0.0012)	0.6664** (0.0010)
**COAD** (269)	0.5944 (0.0014)	0.5944 (0.0044)	0.6034 (0.0012)	0.6347** (0.0027)	0.5823 (0.0028)	0.5779 (0.0058)	0.5950 (0.0025)	0.6292** (0.0028)	0.5961 (0.0010)	0.6120 (0.0018)	0.5975 (0.0007)	0.6348** (0.0014)
**LUSC** (231)	0.4825 (0.0008)	**0.5176 (0.0020)**	0.4830 (0.0008)	0.5140 (0.0029)	0.4858 (0.0012)	0.5175 (0.0033)	0.4898 (0.0012)	**0.5249 (0.0039)**	0.4821 (0.0006)	0.5026 (0.0017)	0.4827 (0.0007)	0.5127** (0.0028)
**SKCM** (198)	0.6262 (0.0005)	0.6314 (0.0016)	0.6327 (0.0005)	0.6352* (0.0016)	0.6190 (0.0012)	0.6310 (0.0018)	0.6275 (0.0012)	**0.6322 (0.0014)**	0.6279 (0.0006)	**0.6371 (0.0009)**	0.6300 (0.0004)	0.6362 (0.0007)
**LUAD** (138)	0.6282 (0.0008)	0.6211 (0.0021)	0.6306 (0.0005)	0.6346** (0.0010)	0.6255 (0.0010)	0.6243 (0.0017)	0.6253 (0.0009)	0.6343** (0.0017)	0.6290 (0.0006)	**0.6312 (0.0011)**	0.6281 (0.0006)	0.6301 (0.0009)
**LAML** (117)	0.6156 (0.0008)	0.5647 (0.0053)	0.6170 (0.0010)	**0.6187 (0.0009)**	0.6022 (0.0017)	0.5792 (0.0031)	0.6071 (0.0018)	**0.6101 (0.0028)**	0.6209 (0.0008)	0.6208 (0.0019)	0.6204 (0.0008)	**0.6215 (0.0009)**
**KIRC** (113)	0.6943 (0.0006)	0.6642 (0.0015)	**0.6970 (0.0005)**	0.6962 (0.0009)	0.6956 (0.0009)	0.6653 (0.0018)	0.6982 (0.0009)	0.7009* (0.0007)	0.6957 (0.0007)	0.6652 (0.0008)	0.6951 (0.0006)	**0.6963 (0.0005)**
**KIRP** (113)	0.7974 (0.0008)	0.7542 (0.0030)	0.7957 (0.0009)	0.8018** (0.0010)	0.7963 (0.0011)	0.7323 (0.0043)	0.7994 (0.0016)	0.8080** (0.0019)	0.7944 (0.0008)	0.7522 (0.0016)	0.7935 (0.0009)	0.8000** (0.0010)
**GBM/LGG** (87)	0.8403 (0.0003)	0.8061 (0.0009)	0.8429 (0.0004)	0.8461** (0.0004)	0.8350 (0.0004)	0.8079 (0.0008)	0.8392 (0.0004)	0.8442** (0.0005)	0.8421 (0.0003)	0.8122 (0.0006)	0.8424 (0.0003)	0.8479** (0.0004)

*Note.* Reported values indicate the average C-index across repeated evaluations, with standard errors shown in parentheses. For each dataset and model, the best-performing method is highlighted in bold, and the second-best method is underlined. The performance improvements of the best method were further statistically assessed using the Wilcoxon signed-rank test against the second-best method; * and ** denote statistical significance at P<0.05 and P<0.01, respectively. The first column lists the cancer datasets and the number of corresponding curated biomarkers.

To evaluate the general applicability of BioExPL across diverse baseline architectures, we considered three survival analysis models: Cox-nnet (Ching *et al.* 2018) and DeepSurv (Katzman *et al.* 2018) have been widely used for survival analysis, and AESurv (Shen *et al.* 2024) was a recently proposed state-of-the-art model. Cox-nnet and DeepSurv are neural network–based proportional hazards models that estimate the log-partial hazard. Cox-nnet uses a single hidden layer, whereas DeepSurv employs a deeper architecture with multiple hidden layers. AESurv extends neural-network–based Cox models by introducing an autoencoder architecture that learns compact latent representations for survival analysis. For all the baseline models, we used the default model architectures reported in their original publications. We applied BioExPL to each model by computing the latent embeddings from the first hidden layer for Cox-nnet and DeepSurv, and as the encoder output for AESurv. In this study, we used Euclidean distance for the knowledge-alignment loss, as it generally showed better predictive performance than cosine similarity (Supplementary Note S2, available as supplementary data at *Bioinformatics* online).

For the benchmark methods to compare the performance with BioExPL, we examined two straightforward schemes: (1) feature filtering and (2) feature weighting (Dinh and Ho 2020). The feature filtering approach reduced the feature dimension by restricting knowledge-guided feature sets only as input to the model. Feature weighting applied the parameter-level L2 regularization to penalize weights associated with non-knowledge features, while leaving knowledge-guided features unregularized. The regularization strength for feature weighting and the knowledge-alignment hyperparameter λ for BioExPL were empirically optimized by minimizing the validation loss.

#### 3.2.1 BioExPL improves the predictive performance

We conducted 5-fold cross-validation and repeated the evaluation 30 times, reporting the average concordance index (C-index) across all folds and repetitions. BioExPL consistently improved the predictive performance of the baseline models across all ten cancer datasets (Table 1). For example, on the LIHC dataset, the knowledge-guided models (i.e. Cox-nnet, DeepSurv, and AESurv coupled with BioExPL) achieved C-index values of 0.6533±0.0014, 0.6553±0.0016, and 0.6474±0.0010, respectively, corresponding to improvements of 3.4%, 4.8%, and 1.8% compared with their baseline models (0.6319±0.0007, 0.6253±0.0015, and 0.6362±0.0006). BioExPL also outperformed feature filtering and feature weighting schemes. Across the ten cancer datasets, Cox-nnet with BioExPL achieved the highest C-index of 0.6684±0.0283, followed by feature weighting (0.6587±0.0303), the baseline model (0.6557±0.0305), and feature filtering (0.6416±0.0254). BioExPL also showed the highest C-index for DeepSurv and AESurv. The performance improvement of BioExPL was statistically validated using the Wilcoxon rank-sum test in the majority of comparisons (P<0.05).

Particularly, BioExPL produced substantial performance gains for LUSC and COAD, with increases of 7.0% and 7.1%, respectively. LUSC has been reported as one of the most challenging cancer types in previous survival analysis studies (Ching *et al.* 2018, Huang *et al.* 2020, Meng *et al.* 2022), often exhibiting C-index values close to 0.5. The observed improvement indicates that BioExPL mitigates this challenge by guiding the model with curated gene associations. COAD is known to be governed by a small number of dominant biological programs that largely influence patient prognosis, as consistently reported by large-scale transcriptomic stratification studies (Joanito *et al.* 2022), pathway-oriented subtype analyses (Komor *et al.* 2018), and tumor microenvironment–focused investigations (Qi and Zhang 2022). This molecular characteristic provides a favorable setting for leveraging curated genes, resulting in more pronounced performance improvements over baseline models.

**Figure 3:**
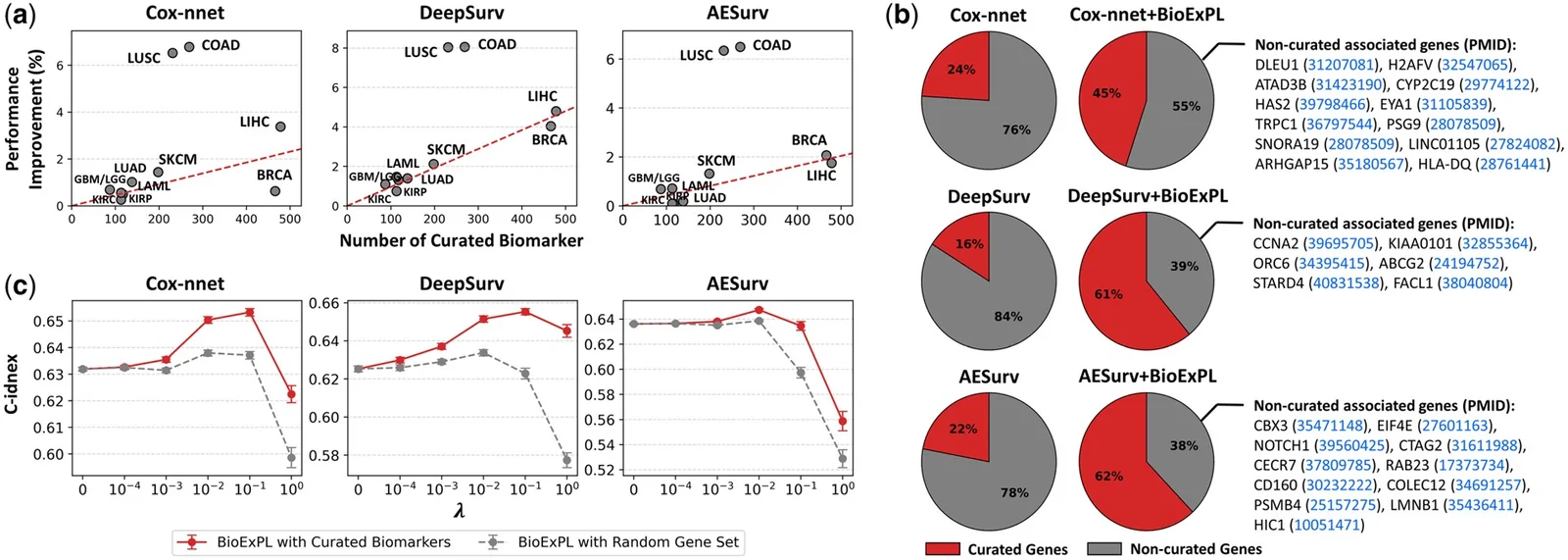
Experimental results on TCGA cancer datasets. (a) Association between the number of curated biomarkers and the performance improvement achieved by BioExPL. Each point represents one cancer cohort, where the *x*-axis denotes the number of curated biomarkers and the *y*-axis indicates the relative performance improvement in C-index (%). The red dashed line shows the linear regression fit, and the Pearson correlation coefficient (*r*) is reported in the main text. The linear regression and Pearson correlation were estimated excluding the outlier cohorts LUSC and COAD, which exhibited particularly substantial performance improvements. (b) Proportion of curated and non-curated genes among the top 100 features identified by baseline and BioExPL-guided models on the LIHC dataset. Top-ranked genes were determined based on feature importance scores computed using Integrated Gradients, averaged across repeated evaluations. Non-curated associated genes refer to genes that are not included in the curated biomarker set but were identified by BioExPL; annotated genes are supported by biological literature, with corresponding PubMed identifiers (PMIDs) shown. (c) Predictive performance of BioExPL using curated biomarkers and random gene sets on the LIHC dataset. The *x*-axis represents the knowledge-alignment regularization parameter λ. C-index values are shown across varying λ for Cox-nnet, DeepSurv, and AESurv.

We observed that the performance of BioExPL increased approximately in proportion to the number of knowledge-guided genes (Fig. 3a). The number of curated biomarkers exhibited statistically significant Pearson correlations with performance improvement, with correlation coefficients of 0.64 (P<0.086), 0.98 (P<0.01), and 0.88 (P<0.01) for Cox-nnet, DeepSurv, and AESurv, respectively. These results may imply that the effectiveness of BioExPL is closely related to the available quantity of the prior knowledge set, so baseline models can be further improved by using richer curated biomarkers. Furthermore, we examined the computational efficiency of BioExPL and observed that it introduces only a marginal computational overhead to each baseline model. A detailed efficiency analysis of BioExPL is provided in Supplementary Note S3, available as supplementary data at *Bioinformatics* online.

#### 3.2.2 BioExPL enhances model interpretability

We evaluated whether BioExPL improves the interpretability of the baseline models. Specifically, we examined the top-100 ranked genes by feature importance in the LIHC dataset, which contained the largest number of knowledge-guided genes, and compared them with the LIHC curated biomarker set. Across all baseline models, BioExPL consistently enhanced the model interpretability, identifying a larger number of curated biomarkers among the top-ranked genes (Fig. 3b). The knowledge-guided models identified 45, 61, and 62 curated biomarkers among the top-100 genes, whereas the corresponding baseline models identified only 24, 16, and 22 curated biomarkers each.

We further examined whether knowledge-guided models can identify potentially associated genes beyond the curated gene set. We searched the top-ranked genes in the biological literature and found that several of them were reported in independent studies supporting their association with LIHC. Specifically, knowledge-guided Cox-nnet identified 12 additional associated genes, while knowledge-guided DeepSurv and AESurv identified 7 and 11 genes, respectively.

### 3.3 Comparative experiment with random gene sets

We additionally examined whether the performance gains of BioExPL are primarily driven by curated biomarkers, rather than to other potential confounding factors. We hypothesized that substituting the curated gene set with a randomly selected gene set would substantially impair the predictive performance of BioExPL. For each cancer set, we replaced the curated gene set with randomly generated gene set. As a result, BioExPL with randomly defined gene sets led to a significant decrease in predictive performance across all cancer datasets (Table 2; P<0.01 in the majority of comparisons). In particular, the average C-index was even lower than that of the corresponding baseline models in the datasets SKCM, LAML, KIRC and KIRP.

**Table 2:** Results of comparative experiments using random gene sets.

	**Cox-nnet**		**DeepSurv**		**AESurv**	
Dataset	BioExPL with random genes	BioExPL	BioExPL with random genes	BioExPL	BioExPL with random genes	BioExPL
LIHC	0.6380±0.0011	**0.6533** ± 0.0014**	0.6338±0.0016	**0.6553** ± 0.0016**	0.6387±0.0008	**0.6474** ± 0.0010**
BRCA	0.6471±0.0012	**0.6499** ± 0.0011*	0.6248±0.0018	**0.6390** ± 0.0015**	0.6593±0.0010	**0.6664** ± 0.0010**
COAD	0.6208±0.0024	**0.6347** ± 0.0027**	0.6000±0.0032	**0.6292** ± 0.0028**	0.6150±0.0013	**0.6348** ± 0.0014**
LUSC	0.5107±0.0038	**0.5140** ± **0.0029**	0.5212±0.0027	**0.5249** ± **0.0039**	0.5099±0.0024	**0.5127** ± **0.0028**
SKCM	0.6255±0.0015	**0.6352** ± 0.0016**	0.6240±0.0010	**0.6322** ± 0.0014**	0.6307±0.0010	**0.6362** ± 0.0007**
LUAD	0.6302±0.0013	**0.6346** ± 0.0010**	0.6335±0.0013	**0.6343** ± **0.0017**	**0.6330** ± **0.0011**	0.6301±0.0009
LAML	0.5989±0.0016	**0.6181** ± 0.0016**	0.5892±0.0030	**0.6101** ± 0.0028**	0.6185±0.0009	**0.6215** ± 0.0009**
KIRC	0.6937±0.0007	**0.6962** ± 0.0009*	0.6920±0.0007	**0.7009** ± 0.0007**	0.6928±0.0006	**0.6963** ± 0.0005**
KIRP	0.7968±0.0012	**0.8018** ± 0.0010**	0.7963±0.0015	**0.8080** ± 0.0019**	0.7931±0.0011	**0.8000** ± 0.0010**
GBM/LGG	0.8449±0.0003	**0.8461** ± 0.0004*	0.8422±0.0004	**0.8442** ± 0.0005**	0.8470±0.0003	**0.8479** ± 0.0004*

*Note*. Results are reported as mean±standard error of the C-index over repeated evaluations. For each comparison, the higher-performing method is highlighted in bold. Statistical significance was assessed using the Wilcoxon signed-rank test, where * and ** denote P<0.05 and P<0.01, respectively.

We also investigated the model performance across varying λ in the LIHC dataset, which contained the largest number of curated biomarkers (Fig. 3c). We observed that predictive performance consistently decreased across all values of λ when random gene sets were used, with more pronounced degradation under stronger knowledge alignment (e.g. $10^{-2}<\lambda$). When BioExPL was applied with curated gene sets, Cox-nnet, DeepSurv, and AESurv achieved performance gains of 3.4%, 4.8%, and 1.8%, respectively, on the LIHC dataset. On the other hand, when using randomly defined gene sets, the corresponding performance gains were only 1.0%, 1.4%, and 0.4%, respectively.

## 4 Discussion

In this study, we proposed a novel biomarker-driven knowledge-guided learning (BioExPL) by incorporating curated prior knowledge. BioExPL guides neural networks to reflect curated knowledge in their latent representations through a knowledge-alignment loss. Across both simulation studies and large-scale survival analysis, BioExPL consistently improved predictive performance and enhanced model interpretability. In the simulation study, we showed that BioExPL can accurately identify both curated and potentially associated features under controlled settings. In the survival analysis with multiple cancer datasets, BioExPL effectively identified the curated biomarkers and further revealed disease-associated genes supported by external biological literature. Our comparative experiment further supported that the observed gains are driven by meaningful biological priors rather than incidental regularization effects. In terms of efficiency, BioExPL incurred only a modest computational overhead, reflecting an implementation that remains computationally efficient.

Despite these overall gains, the effectiveness of BioExPL is still contingent on both the quantity and the quality of the curated knowledge available. In the survival analysis, we observed that BioExPL’s performance improved substantially as more curated biomarkers became available, whereas the gains were relatively modest when only a small set of curated genes was accessible. For instance, BioExPL achieved performance improvements of up to 7% in cancer types with relatively large curated gene sets (e.g. LUSC, COAD, BRCA, and LIHC), while the gains were less than 2% for cancer types with the smallest curated gene sets (e.g. LAML, KIRC, KIRP, and GBM/LGG). In these cancer datasets with smaller curated gene sets, the feature filtering approach resulted in inferior performance compared to the baseline models, suggesting that the curated sets may not be comprehensive enough to fully represent the underlying disease mechanisms. Moreover, the comparative experiment using random gene set priors demonstrated how the quality of the curated gene set influences the extent of performance improvement.

Curated and experimentally validated knowledge—such as biomarker sets, reference feature sets, and expert-defined catalogs—is among the most common and accessible forms of prior information across scientific disciplines. As a model-agnostic and domain-independent learning strategy, BioExPL can be applied to any neural network-based architectures for high predictive power and robust interpretation with minimum computational burden.

## Supplementary Material

btag411_Supplementary_Data

## Data Availability

The datasets analyzed in this study were obtained from The Cancer Genome Atlas (TCGA) through cBioPortal (https://www.cbioportal.org/). The synthetic data and source code used in this study are publicly available at https://github.com/datax-lab/BioExPL.
