# Supplementary material for: Knowledge-guided learning with curated prior genetic biomarkers for robust model interpretation
Source: Bioinformatics. 2026 Aug 21;42(Suppl 2):btag411. doi: 10.1093/bioinformatics/btag411 (PMC13501302; doi:10.1093/bioinformatics/btag411)
Supplement: Supplementary file 1 [file btag411_supplementary_data.pdf]

SUPPLEMENTARY NOTES

Knowledge-Guided Learning with Curated Prior Genetic Biomarkers for Robust Model Interpretation

Beomsu Baek[1], Eunyoung Jang[1], Sai Phani Parsa[1], Youngsoon Kim[2*], and Mingon Kang[1*]

[1] Department of Computer Science, University of Nevada, Las Vegas, Las Vegas, NV 89154, USA

[2] Department of Information and Statistics, Gyeongsang National University, Jinju, Republic of Korea

BB: beomsu.baek@unlv.edu

EJ: eunyoung.jang@unlv.edu

SPP: sai.parsa@unlv.edu

YK: youngsoonkim@gnu.ac.kr

MK: mingon.kang@unlv.edu

*Corresponding authors: Youngsoon Kim and Mingon Kang

Supplementary Notes

Supplementary Note 1: TCGA datasets and curated biomarkers

We used ten cancer cohorts from The Cancer Genome Atlas (TCGA), including Liver Hepatocellular Carcinoma (LIHC), Breast Invasive Carcinoma (BRCA), Colon Adenocarcinoma (COAD), Lung Squamous Cell Carcinoma (LUSC), Skin Cutaneous Melanoma (SKCM), Lung Adenocarcinoma (LUAD), Acute Myeloid Leukemia (LAML), Kidney Renal Clear Cell Carcinoma (KIRC), Kidney Renal Papillary Cell Carcinoma (KIRP), and Glioblastoma Multiforme and Lower Grade Glioma (GBM/LGG). Curated biomarkers for each cancer type were obtained from the Comparative Toxicogenomics Database (CTD), which provides manually curated gene–disease associations supported by extensive biological literature. A brief description of each dataset and the number of identified curated biomarkers are summarized in Table S1.

Table S1. Summary of TCGA cancer datasets.

Cancer type	Description	Sample #	Gene #	Curated biomarker #
LIHC	Liver Hepatocellular Carcinoma	372	20,021	478
BRCA	Breast Invasive Carcinoma	1,099	20,194	466
COAD	Colon Adenocarcinoma	378	19,940	269
LUSC	Lung Squamous Cell Carcinoma	495	20,167	231
SKCM	Skin Cutaneous Melanoma	463	20,167	198
LUAD	Lung Adenocarcinoma	508	20,095	138
LAML	Acute Myeloid Leukemia	163	19,649	117
KIRC	Kidney Renal Clear Cell Carcinoma	534	20,194	113
KIRP	Kidney Renal Papillary Cell Carcinoma	290	20,104	113
GBM/LGG	Glioblastoma Multiforme and Lower Grade Glioma	693	19,777	87

Gene expression (RNA-seq) data were obtained from The Cancer Genome Atlas (TCGA), and curated biomarkers were defined using gene–disease associations from the Comparative Toxicogenomics Database (CTD). Note: Sample # denotes the number of patients, Gene # denotes the number of gene features, and Curated biomarker # indicates the size of the biologically validated biomarker set used as prior knowledge.

Supplementary Note 2: Knowledge-alignment loss using cosine similarity

The proposed knowledge-alignment loss can be formulated using various similarity or distance metrics. In this supplementary note, we present an alternative implementation of the knowledge-alignment loss using cosine similarity and provide the corresponding experimental results. Instead of Euclidean distance, the knowledge-induced representation can be constructed using the sample-wise cosine similarity matrices. Given the knowledge-guided feature matrix $X_K \in \mathfrak{R}^{n\times|K|}$, the knowledge-induced matrix $\widetilde{M}^K \in \mathfrak{R}^{n\times n}$ is defined as

$$\widetilde{M}_{ij}^{K} = \frac{X_K^{(i)} \cdot X_K^{(j)}}{\left\|X_K^{(i)}\right\|_2 \left\|X_K^{(j)}\right\|_2}, \forall i,j \in \{1, \dots, n\}.$$

The latent matrix derived from the learned representations H is computed as

$$\widetilde{M}_{ij}^{H} = \frac{H^{(i)} \cdot H^{(j)}}{\|H^{(i)}\|_2 \|H^{(j)}\|_2}.$$

The knowledge-alignment loss can then be defined by minimizing the sum of squared errors between the two similarity matrices:

$$L_{KAL} = \sum_{(i,j)\in\Omega}\left(\widetilde{M}_{ij}^{K} - \widetilde{M}_{ij}^{H}\right)^2,\ \Omega = \{(i,j)|i < j\}.$$

We conducted survival analysis on the same ten TCGA cancer cohorts using the alternative similarity formulation, following the same experimental protocol described in the main text. Both versions of BioExPL consistently improved predictive performance over the three baseline survival models, with statistically significant improvements observed in the majority of comparisons (Wilcoxon signed-rank test, $p < 0.05$). Interestingly, the relative performance between the two similarity formulations depended on the number of curated biomarkers available in each cohort. For datasets with larger curated biomarker sets (e.g., LIHC and BRCA) the cosine similarity–based formulation achieved higher predictive performance. In contrast, for datasets with relatively small curated biomarker sets (e.g., KIRC, KIRP, and GBM/LGG) the Euclidean distance formulation showed more favorable performance. These observations suggest that, when the curated biomarker feature space is relatively high-dimensional, cosine similarity may more effectively capture the directional relationships among samples

defined by prior knowledge. Conversely, when the curated biomarker dimension is limited, Euclidean distance may provide a more stable measure of sample similarity. Overall, these results demonstrate that the BioExPL is robust to different similarity formulations and can maintain consistent performance improvements across diverse experimental settings.

Table S2. Predictive performance comparison across ten TCGA cancer datasets.

**Dataset**	**Curated biomarker #**	**Cox-nnet**			**DeepSurv**			**AESurv**		
		Baseline	BioExPL (Cosine)	BioExPL (Euclidean)	Baseline	BioExPL (Cosine)	BioExPL (Euclidean)	Baseline	BioExPL (Cosine)	BioExPL (Euclidean)
LIHC	478	0.6319<br>(0.0007)	**0.6565**<br>(0.0013)**	0.6533**<br>(0.0014)	0.6253<br>(0.0015)	**0.6583**<br>(0.0019)**	0.6553**<br>(0.0016)	0.6362<br>(0.0006)	**0.6578**<br>(0.0020)**	0.6474**<br>(0.0010)
BRCA	466	0.6458<br>(0.0013)	**0.6585**<br>(0.0010)**	0.6499*<br>(0.0011)	0.6142<br>(0.0020)	**0.6452**<br>(0.0014)**	0.6390**<br>(0.0015)	0.6529<br>(0.0010)	0.6612**<br>(0.0010)	**0.6664**<br>(0.0010)**
COAD	269	0.5944<br>(0.0014)	0.6332**<br>(0.0031)	**0.6347**<br>(0.0027)**	0.5823<br>(0.0028)	0.6228**<br>(0.0037)	**0.6292**<br>(0.0028)**	0.5961<br>(0.0010)	**0.6398**<br>(0.0030)**	0.6348**<br>(0.0014)
LUSC	231	0.4825<br>(0.0008)	**0.5162**<br>(0.0034)**	0.5140**<br>(0.0029)	0.4858<br>(0.0012)	0.5227**<br>(0.0033)	**0.5249**<br>(0.0039)**	0.4821<br>(0.0006)	0.5030**<br>(0.0020)	**0.5127**<br>(0.0028)**
SKCM	198	0.6262<br>(0.0005)	0.6317**<br>(0.0018)	**0.6352**<br>(0.0016)**	0.6190<br>(0.0012)	**0.6328**<br>(0.0012)**	0.6322**<br>(0.0014)	0.6279<br>(0.0006)	0.6348**<br>(0.0015)	**0.6362**<br>(0.0007)**
LUAD	138	0.6282<br>(0.0008)	**0.6360**<br>(0.0017)**	0.6346**<br>(0.0010)	0.6255<br>(0.0010)	**0.6368**<br>(0.0011)**	0.6343**<br>(0.0017)	0.6290<br>(0.0006)	**0.6342*<br>(0.0017)**	0.6301<br>(0.0009)
LAML	117	0.6156<br>(0.0008)	**0.6188*<br>(0.0012)**	0.6187*<br>(0.0009)	0.6022<br>(0.0017)	0.6073<br>(0.0031)	**0.6101**<br>(0.0028)**	0.6209<br>(0.0008)	0.6209<br>(0.0006)	**0.6215<br>(0.0009)**
KIRC	113	0.6943<br>(0.0006)	**0.6962*<br>(0.0005)**	0.6962*<br>(0.0009)	0.6956<br>(0.0009)	0.6990*<br>(0.0010)	**0.7009**<br>(0.0007)**	0.6957<br>(0.0007)	0.6953<br>(0.0005)	**0.6963<br>(0.0005)**
KIRP	113	0.7974<br>(0.0008)	0.7992<br>(0.0020)	**0.8018**<br>(0.0010)**	0.7963<br>(0.0011)	0.7995<br>(0.0013)	**0.8080**<br>(0.0019)**	0.7944<br>(0.0008)	0.7993**<br>(0.0025)	**0.8000**<br>(0.0010)**
GBM/LGG	87	0.8403<br>(0.0003)	0.8404<br>(0.0003)	**0.8461**<br>(0.0004)**	0.8350<br>(0.0004)	0.8363*<br>(0.0005)	**0.8442**<br>(0.0005)**	0.8421<br>(0.0003)	0.8443*<br>(0.0003)	**0.8479**<br>(0.0004)**

Note: reported values indicate the average C-index across repeated evaluations, with standard errors shown in parentheses. For each dataset and model, the best-performing method is highlighted in bold, and the second-best method is underlined. The performance improvements of BioExPL using cosine similarity and Euclidean distance were statistically assessed using the Wilcoxon signed-rank test against their corresponding baseline models; * and ** denote statistical significance at $p < 0.05$ and $p < 0.01$, respectively. Curated biomarker # denotes the number of curated biomarkers used as knowledge-guided features.

Supplementary Note 3: Computational efficiency of BioExPL

BioExPL relies on sample-wise similarity matrices, which incur a computational cost of $O(n^2)$ for n samples. To mitigate this potential overhead, BioExPL is implemented in a computationally efficient manner. During mini-batch training with batch size b, the computation of the knowledge-alignment loss is performed only within each batch, reducing the per-iteration computational cost from $O(n^2)$ to $O(b^2)$. The knowledge-induced matrix $\widetilde{M}^K$ serves as a fixed reference and is computed only once prior to training. This matrix is reused throughout the optimization process without further updates. Importantly, BioExPL does not modify the architecture of the baseline model and is applied exclusively during training, thereby introducing only minimal additional computational cost and no memory overhead during inference.

We further evaluated the computational overhead introduced by BioExPL using the TCGA survival analysis experiments (Fig. S1). BioExPL introduced only a modest computational overhead across all three baseline survival models. Specifically, for the LIHC dataset, when coupled with BioExPL, Cox-nnet, DeepSurv, and AESurv required 0.0993 ± 0.0002, 0.1001 ± 0.0004, and 0.1131 ± 0.0005 seconds per epoch, respectively. The corresponding baseline Cox-nnet, DeepSurv, and AESurv models required 0.0925 ± 0.0001, 0.0941 ± 0.0001, and 0.1088 ± 0.0001 seconds per epoch. These results correspond to modest computational overheads of 7.3%, 6.3%, and 4.0% for Cox-nnet, DeepSurv, and AESurv, respectively.

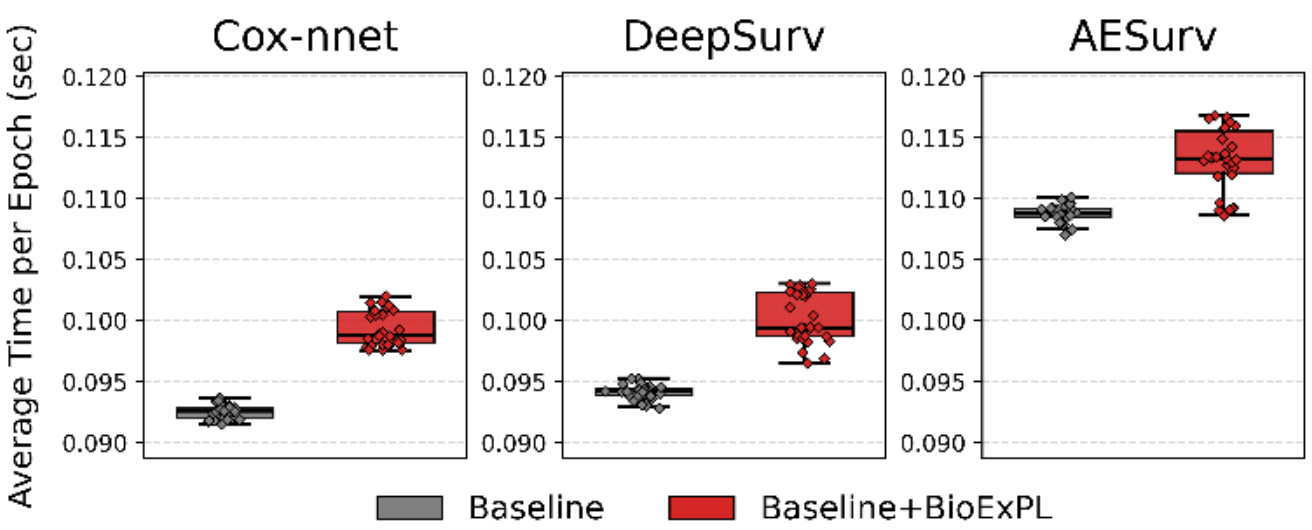

Fig. S1. Comparison of training time between baseline survival models and their BioExPL-guided counterparts on the LIHC dataset. The average training time per epoch (seconds) is shown for Cox-nnet, DeepSurv, and AESurv.
